# Bilateral Hip Joint Hylan G-F 20 Granulomatous Synovitis due to Viscosupplementation Injections

**DOI:** 10.1155/2014/494073

**Published:** 2014-08-25

**Authors:** Patrick Weinrauch, Robert Trigger, George Tsikleas

**Affiliations:** ^1^Brisbane Hip Clinic, QLD, Australia; ^2^School of Medicine, Griffith University, QLD, Australia; ^3^North Coast Medical Centre, Byron Bay, NSW, Australia; ^4^Sullivan Nicolaides Pathology, QLD, Australia

## Abstract

We present the diagnosis of bilateral granulomatous inflammation of the hip joints associated with Hylan G-F 20 viscosupplementation injections. Clinicians recommending therapeutic Hylan injections for the management of hip arthritis should maintain clinical awareness regarding this potential complication.

## 1. Introduction

Viscosupplementation injections for the management of osteoarthritis of the hip have been demonstrated to be both safe and effective [1–9]. Acute injection related synovitis is observed in approximately 5–10% of patients undertaking this treatment, but these reactions are characteristically transient without longer-term clinical effects [10–15]. Chronic granulomatous inflammatory synovial reactions associated with Hylan injections have however been described within the knee joint as an infrequent occurrence [11, 16, 17]. To our knowledge, we present the first description of Hylan induced granulomatous inflammation within the hip joint.

## 2. Case Presentation

A 52-year-old male presented with bilateral hip osteoarthritis secondary to cam type femoroacetabular impingement, particularly affecting the right hip joint. He described symptoms spanning a period of a number of years, slowly progressive in nature with typical features associated with osteoarthritic degeneration. The patient was otherwise well and besides intermittent analgesics and glucosamine was taking no other medications. No other joint pathologies were noted with the exception of previous subacromial bursitis of the right shoulder, successfully treated by arthroscopic decompression.

**Figure 1:**
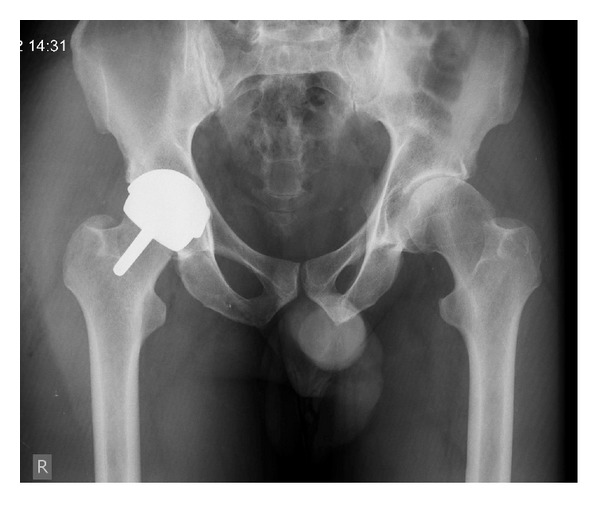
Postoperative radiograph after right Birmingham Hip Resurfacing. Left hip demonstrates features consistent with cam type femoroacetabular impingement and secondary osteoarthritis.

Previous management for his right hip had included central compartment arthroscopic debridement seven months previously. In an attempt to optimise his joint preservation therapies, the patient had also undertaken injectable therapies. A single 2 mL Synvisc (Hylan G-F 20, Genzyme Biosurgery, Ridgefield, USA) injection had initially been administered to the patient four months earlier and approximately six weeks later he had been treated with adipose derived stem cell therapy injection. On the basis of the severity of his degenerative joint disease on both clinical and radiographic grounds, the patient was recommended for definitive treatment by Birmingham Hip Resurfacing Arthroplasty (Figure 1).

**Figure 2:**
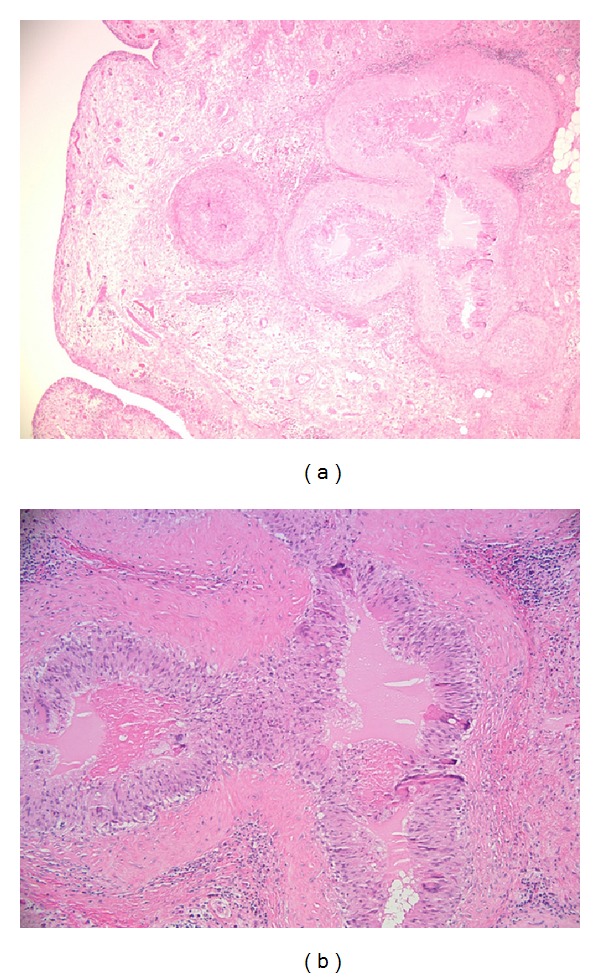
(a) Low power H&E stain of synovial specimen demonstrating granulomatous inflammation. (b) High power image of (a) H&E stain. Central amorphous material demonstrated.

Operative findings at time of surgical intervention for his right hip resurfacing included macroscopically florid hypertrophic synovitis, substantially beyond that typically seen with osteoarthritic degeneration. Biopsies were accordingly taken which demonstrated histological evidence of chronic granulomatous change (Figures 2(a) and 2(b)). No bacteria were identified on Gram stain and no crystals were detected. Cultures remained negative and specific staining and histological evaluation conducted to evaluate a cause for the granulomatous synovitis failed to identify mycobacteria or fungal elements. The patient's recovery after his resurfacing procedure was unremarkable, with a good clinical result in terms of pain relief and function. Despite the finding of florid synovitis within the joint at time of arthroplasty, the postsurgical recovery appeared to be unaffected. The precise cause for the granulomatous synovitis observed within the hip joint at this stage was not determined but was felt possibly to be due to a reaction secondary to the adipose derived stem cell therapy.

Twelve months after his right hip resurfacing, the patient described increasingly problematic symptoms due to left hip degenerative joint disease, interfering with function. X-rays demonstrated significant arthritic change with radiographic loss of articular joint space. The decision was made at this time to treat the left hip by a single Hylan G-F 20 viscosupplementation injection (Synvisc 2 mls). The initial response after the injection was unremarkable; however, within seven days, the patient described increasing discomfort. The symptoms at this stage were consistent with articular hip irritation; however, he remained ambulant and systemically well without signs of infection. The clinical picture was consistent with an acute local reactive synovitis and recommendations made for continued observation. Accordingly, the acute features seen about the time of injection settled however on the basis of continued chronic joint irritability and significant arthritis on radiographic grounds; a decision was made to proceed with a Birmingham Hip Resurfacing two months after his left hip Hylan injection. At time of surgical intervention, again macroscopic florid hypertrophic synovitis was observed. Histology specimens upon the left hip similarly demonstrated chronic synovitis with granulomatous inflammation consistent with those observed on the right hip one year previously. Culture growth remained negative and no crystals or mycobacterial or fungal elements were identified. Clinical recovery after left resurfacing arthroplasty was also unremarkable, with clinical results apparently unaffected by the finding of florid synovitis at time of surgery.

**Figure 3:**
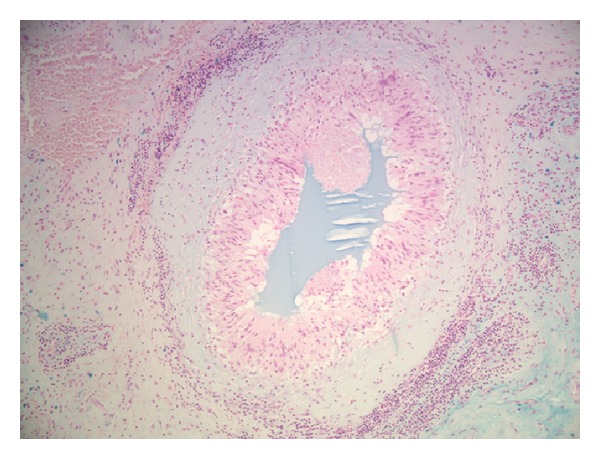
Alcian blue stain of central material consistent with previously injected Hylan G-F 20.

**Figure 4:**
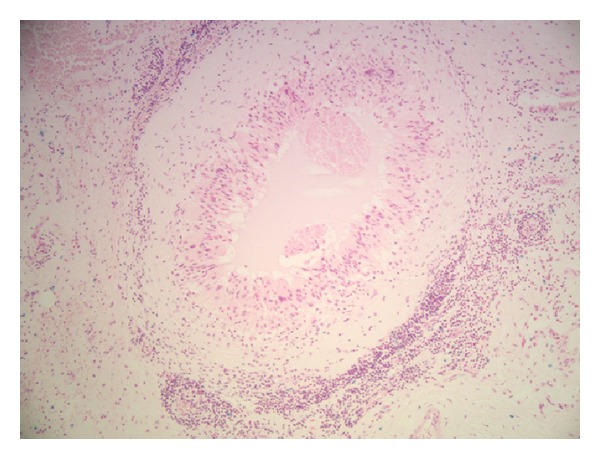
Alcian blue stain after treatment of specimen by hyaluronidase. Lack of staining in centre of granuloma indicative of enzymatic digestion.

On the basis of the operative histology findings of bilateral granulomatous inflammation, the patient was evaluated for a potential underlying systemic or infective pathology, including but not limited to mycobacterium serology (Quantiferon TB Gold assay), cyclic citrullinated peptide (CCP) antibodies, rheumatoid factor (RF) latex, and antineutrophil cytoplasmic antibodies (ANCA). These evaluations revealed no identifiable evidence of systemic or infective cause. Further evaluation of the histological specimens was subsequently requested, specifically to evaluate a potential association with the previous conducted Hylan injections. Alcian blue stain demonstrated Hylan material centrally located within the granulomas with evidence of predigestion of the material when treated with hyaluronidase (Figures 3 and 4). On the basis of these findings, together with the clinical history and macroscopic appearance at time of surgery in both hips, the diagnosis of chronic granulomatous inflammation, secondary to Hylan G-F 20 (Synvisc) injections, was diagnosed.

## 3. Discussion

Granulomatous inflammation associated with viscosupplementation in the knee joint was described by Chen et al. [16] in six patients with chronically inflamed synovium with histiocytic foreign body giant cell reactions observed on microscopy. Central within the granulomas, acellular amorphous material was identified by Alcian blue stain which was subsequently removed by hyaluronidase digestion. The authors concluded that the granulomatous inflammation was secondary to the injected Hylan. Granulomatous inflammation within the knee has also been described by Zardawi and Chan [11].

Sasaki et al. [18, 19] demonstrated the subcutaneous and intramuscular induction of a delayed foreign body granulomatous inflammation in Guinea pig and rabbit models with the conclusion that Hylan may also potentially induce unfavourable soft tissue reactions in humans, contradicting previous reports of favourable biocompatibility. Waddell et al. [17] demonstrated hyaluronate granulomas in 5.9% of patients undergoing total knee replacement who had previously been treated with Hylan G-F 20 injections. Half of the patients who demonstrated histological evidence of hyaluronate granulomata had previously experienced acute local reactions within 30 days of administration of the injection. The authors questioned the clinical significance of synovial hyaluronate granulomas within the knee joint, although only a limited number of patients were within the affected cohort.

Hylan viscosupplementation injections are a relatively recent therapeutic strategy for the management of hip joint osteoarthritis in comparison to their use in knee pathology. They remain an effective management strategy, particularly for patients who are not suitable for definitive surgical intervention on clinical or other grounds. The most common side effect, being a transient acute postinjection local joint irritation, can be managed by symptomatic measures alone. To our knowledge, this is the first description of hyaluronate induced chronic granulomatous inflammation within the hip joint due to viscosupplementation injections. Clinicians recommending hyaluronate injections for the management of hip arthritis should maintain clinical awareness regarding this potential complication.

## References

[1] Brocq O, Tran G, Breuil V, Grisot C, Flory P, Euller-Ziegler L (2002). Hip osteoarthritis: short-term efficacy and safety of viscosupplementation by hylan G-F 20. An open-label study in 22 patients. *Joint Bone Spine*.

[2] Caglar-Yagci H, Unsal S, Yagci I, Dulgeroglu D, Ozel S (2005). Safety and efficacy of ultrasound-guided intra-articular hylan G-F 20 injection in osteoarthritis of the hip: a pilot study. *Rheumatology International*.

[3] Migliore A, Bizzi E, Massafra U (2012). The impact of treatment with hylan G-F 20 on progression to total hip arthroplasty in patients with symptomatic hip OA: a retrospective study. *Current Medical Research and Opinion*.

[4] Migliore A, Tormenta S, Martin LSM (2005). Open pilot study of ultrasound-guided intra-articular injection of hylan G-F 20 (Synvisc) in the treatment of symptomatic hip osteoarthritis. *Clinical Rheumatology*.

[5] Migliore A, Tormenta S, Martin Martin LS (2006). The symptomatic effects of intra-articular administration of hylan G-F 20 on osteoarthritis of the hip: clinical data of 6 months follow-up. *Clinical Rheumatology*.

[6] Migliore A, Tormenta S, Massafra U (2008). Intra-articular administration of hylan G-F 20 in patients with symptomatic hip osteoarthritis: tolerability and effectiveness in a large cohort study in clinical practice. *Current Medical Research and Opinion*.

[7] Rennesson-Rey B, Rat A-C, Chary-Valckenaere I (2008). Does joint effusion influence the clinical response to a single Hylan GF-20 injection for hip osteoarthritis?. *Joint Bone Spine*.

[8] van den Bekerom MPJ, Lamme B, Sermon A, Mulier M (2008). What is the evidence for viscosupplementation in the treatment of patients with hip osteoarthritis? Systematic review of the literature. *Archives of Orthopaedic and Trauma Surgery*.

[9] Spitzer AI, Bockow BI, Brander VA (2010). Hylan G-F 20 improves hip osteoarthritis: a prospective, randomized study. *The Physician and Sportsmedicine*.

[10] Hamburger M, Settles M, Teutsch J (2005). Identification of an immunogenic candidate for the elicitation of severe acute inflammatory reactions (SAIRs) to hylan G-F 20. *Osteoarthritis and Cartilage*.

[11] Zardawi IM, Chan I (2001). Synvisc perisynovitis. *Pathology*.

[12] Goomer RS, Leslie K, Maris T, Amiel D (2005). Native hyaluronan produces less hypersensitivity than cross-linked hyaluronan. *Clinical Orthopaedics and Related Research*.

[13] Morton AH, Shannon P (2003). Increased frequency of acute local reaction to intra-articular Hylan G-F 20 (Synvisc) in patients receiving more than one course of treatment. *The Journal of Bone and Joint Surgery A*.

[14] Goldberg VM, Coutts RD (2004). Pseudoseptic reactions to hylan viscosupplementation : diagnosis and treatment. *Clinical Orthopaedics and Related Research*.

[15] Waddell DD (2003). The tolerability of viscosupplementation: low incidence and clinical management of local adverse events. *Current Medical Research and Opinion*.

[16] Chen AL, Desai P, Adler EM, Di Cesare PE (2002). Granulomatous inflammation after hylan G-F 20 viscosupplementation of the knee: a report of six cases. *The Journal of Bone & Joint Surgery A*.

[17] Waddell DD, Beyer A, Thompson TL (2014). No conclusive evidence that histologically found granulomas and acute local reactions following hylan G-F 20 injections are related or have clinical significance. *The Journal of Knee Surgery*.

[18] Sasaki M, Miyazaki T, Nakamura T, Takahashi T, Miyauchi S, Iwata H (2004). Immunogenicity of hylan g- f 20 in Guinea pigs and mice. *The Journal of Rheumatology*.

[19] Sasaki M, Miyazaki Y, Takahashi T (2003). Hylan G-F 20 induces delayed foreign body inflammation in guinea pigs and rabbits. *Toxicologic Pathology*.

